# Mechanical Properties of Protein-Based Hydrogels Derived from Binary Protein Mixtures—A Feasibility Study

**DOI:** 10.3390/polym15040964

**Published:** 2023-02-15

**Authors:** Sandra Haas, Jürgen Hubbuch

**Affiliations:** Institute of Process Engineering in Life Sciences, Section IV: Biomolecular Separation Engineering, Karlsruhe Institute of Technology (KIT), Fritz-Haber-Weg 2, 76131 Karlsruhe, Germany

**Keywords:** protein-based hydrogel, visible light-induced photopolymerization, elastin-like protein, BSA, casein, α-amylase

## Abstract

Hydrogels based on natural polymers such as proteins are considered biocompatible and, therefore, represent an interesting class of materials for application in the field of biomedicine and high-performance materials. However, there is a lack of understanding of the proteins which are able to form hydrogel networks by photoinduced dityrosine crosslinking as well as a profound knowledge of the formed network itself and the mechanisms which are responsible for the resulting mechanical properties of such protein-based hydrogels. In this study, casein, bovine serum albumin, α-amylase, and a hydrophobic elastin-like protein were used to prepare binary protein mixtures with defined concentration ratios. After polymerization, the mechanical properties of the resulting homopolymeric and copolymeric hydrogels were determined using rheological methods depending on the protein shares used. In additional uniaxial compression tests, the fracture strain was shown to be independent of the protein shares, while hydrogel toughness and compressive strength were increased for protein-based hydrogels containing casein.

## 1. Introduction 

Protein-based hydrogels may help to meet the increasing need for non-petroleum-based specialized materials, as they are based on biological renewable resources, have a good cyto-/biocompatibility, and show a high extent of biodegradability [1,2]. Additionally, since some proteins can be expressed as recombinant polymers with high monodispersity and with the precise control and adaptability of their amino acid sequences, they can be customized to the needs of their potential specific application [3]. Since proteins can undergo stimulus-dependent conformational changes, biomaterials. with increasingly complex functions and improved functional properties can be designed [4]. In addition to naturally occurring proteins, artificially designed proteins such as elastin-like proteins (ELPs) can be used as a raw material source for hydrogels proposed for applications in drug delivery [5], tissue engineering [6,7], or in three-dimensional (3D) printing [8,9]. This class of proteins is based on the repetitive core amino acid sequence Val-Pro-Gly-Xaa-Gly of mammalian tropoelastin—with Xaa being any amino acid besides proline—and with mechanical properties comparable to those of natural elastin [10,11,12,13]. The aim of the presented study is thus to use proteins as renewable resources and sources for the generation of new hydrogel materials.

Each type of protein, whether it is a naturally occurring or engineered protein, has particular characteristics in terms of its structure due to specific inter- and intramolecular interactions. Protein-based hydrogels can be prepared either by physical or chemical crosslinking approaches or by a combination of both [14]. One of these hydrogelation methods is based on the crosslinking of phenolic hydroxy groups, which are naturally present in tyrosine, to form dityrosines under mild reaction conditions. This processing pathway can be mediated by a ruthenium-containing photoinitiator which is induced by visible light [15,16,17] and has been applied to several proteins, including bovine serum albumin (BSA), casein, tyrosine-enriched gelatin, maltose-binding protein, I27, protein L, anegen, ELPs, and silk [18,19,20,21,22,23,24,25,26,27,28,29]. An alternative photoinitator system would be riboflavin, which is limited by a slower crosslinking speed and efficiency and is thus less favorable with regard to potential applications in 3D printing or for large volumes [29,30]. However, the main challenge in the development of protein-based hydrogels remains the insufficient understanding of the protein characteristics and polymerization conditions in relation to the resulting mechanical properties of the hydrogels; the limited material availability of artificial protein constructs and the drawback of many influencing factors on the resulting mechanical properties of the hydrogels hinders the design of hydrogels with specific desired mechanical properties.

To shed some light on this question, we conducted the following feasibility study. We propose the formation of copolymeric hydrogels by using binary protein mixtures for crosslinking with the aim of combining the mechanical properties of different homopolymeric protein-based hydrogels. For this purpose, copolymeric hydrogels are made from binary mixtures of three different commercially available proteins (the globular protein BSA, the enzyme α-amylase, and the conjugated casein) and a hydrophobic elastin-like protein construct that is artificially designed. The resulting mechanical properties of the homo- and copolymeric protein-based hydrogel are evaluated in terms of its structural strength, elasticity, compressive fracture strain, compressive strength, and toughness.

## 2. Materials and Methods

### 2.1. Buffer Preparation

The formulation of the buffer solutions was (1) a 20 mM sodium phosphate buffer (SPB) with 4 M urea and (2) a 96 mM multi-component buffer (MCB) consisting of 47 mM N-[tris(hydroxymethyl)methyl]-3-aminopropanesulfonic acid (TAPS), 11 mM 3-morpholino-2-hydroxypropanesulfonic acid (MOPSO), 38 mM sodium citrate, and 4 M urea. All buffers were prepared with ultrapure water (PURELAB Ultra, ELGA LabWater, LaneEnd, UK) and were pH-adjusted to pH 8 using a 4 M sodium hydroxide solution. The buffers were filtered through a 0.45 µm cellulose acetate membrane (Pall Corporation, New York, NY, USA) before use. 

### 2.2. Photoinitiator and Co-Factor 

The photoinitiator tris(2,2′-bipyridyl)dichlororuthenium(II) hexahydrate (Ru(bpy)_3_Cl_2_·6H_2_O) was prepared in the corresponding buffer to a concentration of 5 mM and stored at 4 °C. The electron acceptor ammonium persulfate (APS, chemical formula: (NH_4_)_2_S_2_O_8_) was prepared with a concentration of 2 M in the corresponding buffer, stored as aliquots at −20 °C, and thawed directly prior to usage.

### 2.3. Elastin-like Protein Production 

Following the nomenclature introduced by Meyer et al. [31], the used hydrophobic ELP is referred to as ELP[V2Y-45] with the guest residues valine and tyrosine in a 2:1 ratio and with a total of 45 repetitions of the pentapeptide sequence. Fermentation and purification were performed using the inverse transition cycling (ITC) process previously described [32]. Briefly, the homogenized and subsequently centrifuged *Escherichia coli* lysate was resuspended in a buffer containing 4 M urea to dissolve inclusion bodies containing the ELP constructs. Insoluble contaminants were removed by another round of centrifugation before the addition of 0.4 M ammonium sulfate to precipitate ELP[V2Y-45], which was separated from soluble contaminants by subsequent centrifugation. Subsequently, the pellet of the high salt precipitation was resuspended in ultrapure water. As the hydrophobic ELP[V2Y-45] reversibly aggregated in water at room temperature, two temperature-dependent centrifugation steps were applied in water to remove contaminants (referred to as one cycle of ITC), and the pellet of the hot spin centrifugation finally formulated into 20 mM SPB containing 4 M urea by its resuspension. 

### 2.4. Preparation of the Protein Stock Solutions

Four different proteins were used in this study, a hydrophobic ELP construct, casein, bovine serum albumin (BSA), and α-amylase. Casein (EMD Millipore Corporation, Billerica, MA, USA) was dissolved in the respective buffer at a concentration of 130 mg/mL using a dual asymmetric centrifuge (DAC, SpeedMixer^®^ DAC 150.1 FVZ-K, Hauschild GmbH & Co. KG, Hamm, Germany) at 2500 rpm, while α-amylase (*Bacillus sp.*, A4862, Merck KGaA, Darmstadt, Germany) was purchased as a liquid formulation. Dialysis was performed for the rebuffering of α-amylase and for the purification of casein (e.g., by removing production buffer salts) using SnakeSkin™ dialysis tubing (Thermo Fisher Scientific, Waltham, MA, USA) with a 10 kDa molecular weight cut-off and a 100-fold buffer excess. Two buffer exchanges were performed, the first after > 2 h and the second after >2 more hours. BSA was prepared in the respective buffer solution in the DAC at 2500 rpm directly prior to use. 

If necessary, Vivaspin^®^ ultrafiltration units (Sartorius Stedim Biotech, Göttingen, Germany) with a molecular weight cut-off of 10 kDa were used according to the manufacturer’s recommendation to reach higher stock solution concentrations. Protein concentrations were determined with a NanoDrop 2000c UV-Vis spectrophotometer (Thermo Fischer Scientific, Waltham, MA, USA) using the extinction coefficients ε_BSA,280 nm_ = 0.67 L/(g·cm) [33], ε_α-Amylase,280 nm_ = 2.60 L/(g·cm) [34], ε_ELP[V2Y-45],280 nm_ = 0.799 L/(g·cm) [32], and ε_Casein,280 nm_ = 0.73 L/(g*cm) [22].

### 2.5. Hydrogel Formation

Hydrogels with a total protein concentration of 80 mg/mL composed of a single protein (homopolymeric hydrogels) or a binary protein mixture (copolymeric hydrogels), 0.25 mM Ru(bpy)_3_Cl_2_ and 100 mM APS were prepared as previously described [22]. Briefly, this was performed through the following steps: (1) Formulation buffer, protein, and photoinitiator stock solutions were mixed in the DAC. Another round of mixing in the DAC followed the addition of the APS stock solution. (2) The precursor solution was transferred into a cylindrical mold (diameter 10 mm, height 3 mm). (3) The mold was irradiated for 5 min from atop and below using a blue emitter (LZ4-00B208, LED Engin Inc, San Jose, CA, USA). (4) Polymerized hydrogels were then stored in their formulation buffer for 7 d prior to analytics with a 100-fold liquid excess to monitor the swollen state. 

### 2.6. Oscillatory Rheometry

The linear viscoelastic region (LVR) of polymerized hydrogels was determined by amplitude sweeps (angular frequency ω = 1 and 25 rad·s^−1^, shear stress τ = 5–10.000 Pa, number of replicates n = 2), followed by frequency sweeps within the LVR (τ = 10 Pa, ω = 1–25 rad·s^−1^, n = 3) on a Physica MCR 301 plate rheometer equipped with the plate-plate geometry PP10 (all Anton Paar GmbH, Graz, Austria) to obtain the mean plateau value of the storage modulus G′ and loss factor tan δ. 

### 2.7. Uniaxial Compression Tests

Uniaxial compression tests were performed on a universal testing machine (zwickiLine Z0.5TN, ZwickRoell GmbH & Co. KG, Ulm, Germany) equipped with a stainless-steel compression platen and the Xforce HP 100 N load cell (both ZwickRoell GmbH & Co. KG). Swollen hydrogel discs were compressed with a uniform velocity of 2 mm/min until sample breakdown to determine the fracture strain ε_max_ = (L − L_0_)/L_0_, with L_0_ representing the sample length at a pre-force of 0.2 N and L representing the sample length at sample fracture; for compressive strength σ_max_ = F/A_0_, F represents the applied force at sample fracture and A_0_ represents the unstressed cross-sectional area, and the hydrogel toughness by integrating the stress–strain curves until sample failure in relation to the uncompressed hydrogel volume. 

## 3. Results 

### 3.1. Homopolymeric Protein-Based Hydrogels

**Figure 1:**
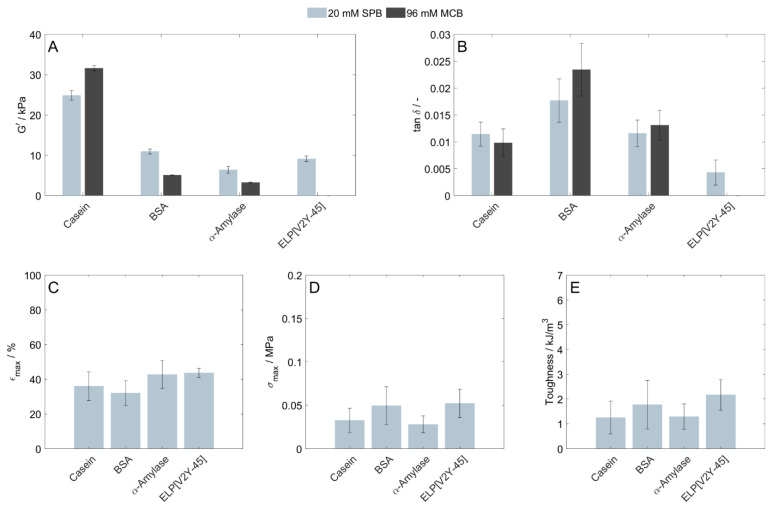
Characterization of protein-based hydrogels consisting of 80 mg/mL of a single protein prepared in 20 mM SPB (light blue) or 96 mM MCB (black). (**A**) Hydrogel storage modulus (G′) and (**B**) Loss factor (tan δ) related to the used protein and buffer (n = 3), as determined by rheometric measurements. Uniaxial compression tests were performed with hydrogels prepared in 20 mM SPB to determine their (**C**) Fracture strain, (**D**) Compressive strength, and (**E**) Toughness.

In order to evaluate their suitability to form homopolymeric protein-based hydrogels, four different proteins with distinct characteristics (see Table 1) were crosslinked, and the resulting mechanical properties of the hydrogels, such as their structural strength and elasticity, were determined. Technically, as casein consists of four different subunits, casein-based hydrogels could be referred to as copolymers; however, for the ease of simplification with regard to the subsequent use of binary protein mixtures, we consider the obtained casein-based as a homopolymeric network within this manuscript. The homopolymeric hydrogels were polymerized by the visible-light-induced crosslinking of a precursor solution containing 80 mg/mL of only one type of protein per formulation. Each protein was prepared in two buffer systems—with the exception of ELP[V2Y-45]—which was prepared in SPB only due to limited material availability. The structural strength of the hydrogels was evaluated in terms of storage modulus G′ (Figure 1A), while elasticity was evaluated on the basis of loss factor (tan δ = G″/G′) with a loss factor of 0 corresponding to ideal elastic behavior (Figure 1B). Both parameters were determined using frequency sweeps, with shear stress selected within the linear viscoelastic range. For all protein stock solutions and buffers, the gel-like behavior of the previously photopolymerized hydrogel networks was confirmed by G′ to be greater than G″ over the whole amplitude range applied. In contrast to the hydrogels prepared from the other proteins investigated, casein hydrogels prepared in SPB developed visible cracks, which did not impede the overall disc integrity during or shortly after polymerization before being transferred to the storage solution. The structural strength of casein hydrogels increased by 27% when prepared in MCB instead of SPB, while for BSA and α-amylase hydrogels, it decreased by 54% (BSA) and 49% (α-amylase). In the same context, elasticity was increased for casein hydrogels by 14%, while there was an opposite trend for hydrogels prepared in BSA (−33%) or α-amylase (−13%). 

When prepared in SPB, α-amylase, BSA, and ELP hydrogels had a storage modulus between 6.4 ± 0.8 kPa (α-amylase) and 10.9 ± 0.6 kPa (BSA), while hydrogels from the conjugated protein casein had the highest storage modulus (24.8 ± 1.2 kPa), and thus, the structural strength of all protein-based hydrogels were tested. Regarding elasticity, BSA hydrogels were the least elastic (tan δ = 0.017 ± 0.004), while hydrogels obtained from casein (tan δ = 0.011 ± 0.002) and α-amylase (tan δ = 0.012 ± 0.002) had comparable elasticity, and the hydrogel prepared for the artificially designed elastin-like protein exhibited the highest elasticity with tan δ = 0.004 ± 0.002. Additional information about the mechanical properties of the protein hydrogels was obtained by uniaxial compression tests. For these analyses, we focused on SPB as the more common buffer system. With regard to the fracture strain (all in the range between 32 ± 7 and 44 ± 3%), compressive strength (all in the range between 0.028 ± 0.010 and 0.052 ± 0.016 MPa) and hydrogel toughness (all in the range between 1.25 ± 0.66 and 2.16 ± 0.62 kJ/m³), and taking the standard deviations into account, no differences were obtained for the investigated proteins (Figure 1C–E). More profound statements about statistical significance would need an increased number of replicates per condition.

### 3.2. Rheological Properties of Copolymeric Hydrogels

**Figure 2:**
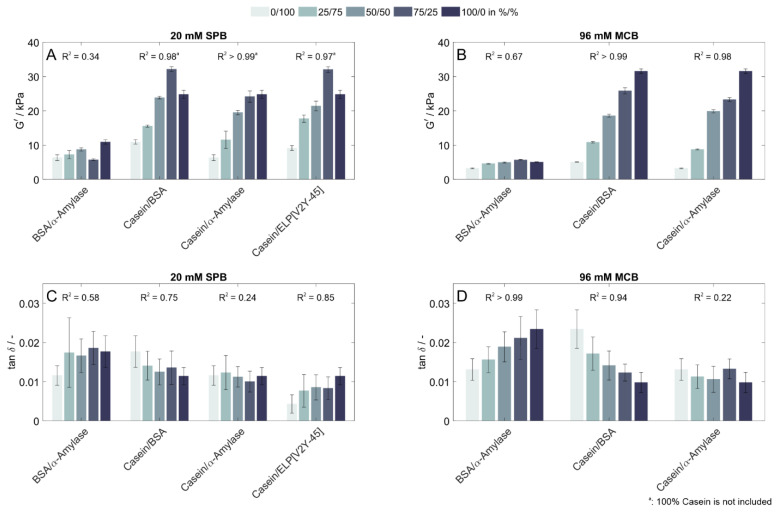
Rheological characterization of hydrogels containing different protein shares as binary mixtures. (**A**) Storage modulus (G′) and (**C**) loss factor (tan δ) of hydrogels prepared and stored in 20 mM SPB, (**B**) G′, and (**D**) Tan δ of hydrogels prepared and stored in 96 mM MCB determined by frequency-dependent oscillatory shear rheology (n = 3). Binary protein mixtures of casein and ELP[V2Y-15] were only prepared in 20 mM SPB and thus not determined in a 96 mM MCB buffer.

To investigate the resulting mechanical properties of copolymeric protein-based hydrogels as a function of the different protein shares, binary mixtures of the proteins were polymerized and analyzed. Hydrogels prepared with 100% casein in SPB developed visible cracks during or shortly after polymerization, which were lower in extent and number for mixtures containing 25% of a second protein and were not found by visual inspection for mixtures containing 50% casein or less. Three findings can be highlighted regarding the structural strength of copolymeric hydrogels prepared from binary mixtures (Figure 2A,B). First, the storage modulus, and thus, the structural strength of copolymeric hydrogels consisting of mixtures of BSA and α-amylase remained in a comparable range: a trend was found for both buffer conditions tested. Second, for two proteins that had higher deviations in their structural strength when prepared as homopolymeric hydrogels—in this case, the combination of casein and another of the proteins studied—a linear correlation (coefficient of determination R^2^ > 0.97) of their structural strength as a function of the protein share was found, when the 100% casein condition was excluded for samples prepared in SPB. Third, the G′ of a hydrogel made from 75% casein and either 25% BSA or ELP[V2Y-45] decreased by 22% compared to a hydrogel made of 100% casein when being prepared in SPB. When comparing it to hydrogel prepared with 25% α-amylase and 75% casein, the G′ of the 100% casein hydrogel still slightly increased by 3%. However, in all cases, this did not follow the linear increase in G′ observed for the increasing amount of casein until then. Interestingly, when these same protein combinations were prepared in a different solution (MCB), G′ linearly increased with increasing casein content up to 100% casein. 

In terms of elasticity (Figure 2C,D), as mentioned earlier, BSA was the least elastic single protein formulation in both buffer conditions, especially when prepared and stored in MCB. Although most sample values were within standard deviations of the tan δ values, copolymers prepared with BSA in combination with either casein or α-amylase showed a fair correlation in SPB (R^2^ = 0.58 (BSA/α-amylase)—0.74 (BSA/casein)) but a strong correlation between their elasticity when being prepared in MCB (R^2^ > 0.94). For copolymers consisting of ELP[V2Y-45]—the protein with the most elastic of all analyzed homopolymeric networks—and casein, a similar correlation was found (R^2^ = 0.85). For copolymers obtained from mixtures of casein and α-amylase with the comparable elasticity of their homopolymers, a low correlation was found (R^2^ < 0.24) in both buffer conditions investigated. 

### 3.3. Uniaxial Compression of Copolymeric Hydrogels

**Figure 3:**
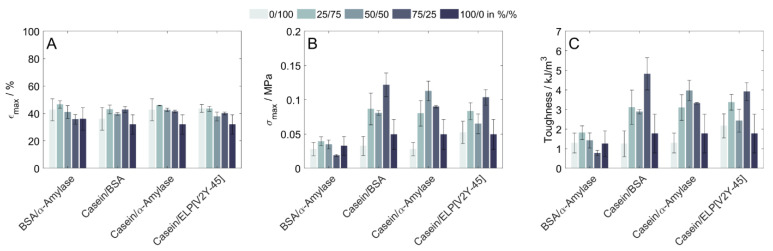
Mechanical characterization of hydrogels containing different protein shares as binary mixtures determined by uniaxial compression. (**A**) Fracture strain, (**B**) Compressive strength, and (**C**) Toughness were determined for hydrogels prepared and stored in 20 mM SPB (n = 3).

In order to obtain more information about the resulting hydrogels, an uniaxial compression was performed for all protein combinations prepared and stored in SPB to evaluate additional mechanical properties. For all tested conditions, the fracture strain was between 32 ± 7% (100% casein) and 47 ± 3% (25% BSA/75% α-amylase), with no clear trend in the data sets (Figure 3A). At the same time, compressive strength and toughness were found to be dependent on the used protein shares (Figure 3B,C). While for BSA/α-amylase, the compressive strength and toughness of all mixtures deviated within their standard deviations for both properties, these properties were increased for hydrogels being prepared as copolymers from a mixture of casein and any other of the investigated proteins compared to the respective homopolymers. In numbers, the maximum increase was observed for mixtures of casein and BSA, with a compressive strength increased by 166% and toughness increased by 149% (75% casein/25% BSA compared to 100% BSA), respectively by 75% and 76% (compressive strength and toughness of 75% casein/25% BSA compared to 100% casein). 

## 4. Discussion

### 4.1. Buffer Components

In order to analyze the potential influence of different buffer mediums on the protein-based hydrogels, in addition to a simple standard phosphate buffer for protein solutions (SPB), we also used a more complex MCB formulation. The buffer components MOPSO and TAPS used in the MCB are known to stabilize the native structure of BSA [42,43] and, thus, possibly stabilize the second protein investigated with an ordered tertiary structure (α-amylase) as well. This may result in a lower crosslinking density due to a lower degree of protein unfolding which was previously shown to be critical for structural strength [22]. The third buffer component, sodium citrate, is known to increase the solubility of casein [44,45], which may increase the surface availability of potential crosslinking sites. As previously described [22], the visible crack formation directly after polymerization for hydrogels containing a casein share ≥75% may thus be related to the non-stabilizing buffer formulation conditions, which may also explain the lower structural strength of homopolymeric casein hydrogels prepared in SPB compared to the copolymers formed with a 75% casein share in SPB. In addition to the different buffer components, the MCB formulation has higher ionic strength, as evidenced by the fact that a total of 96 mM of salt was were instead of 20 mM in the case of the SPB. This affects the swelling behavior and, thus, mechanical properties of the hydrogels, as a higher ionic strength leads to more ionic interactions between mobile ions and fixed charges inside the hydrogels [46]. It was further found that the oxygen concentration in the formulation had an effect on dityrosine crosslinking [17]. By using similar sample preparation pathways, a centrifugal mixing method known for low air entrainments, and consistent concentrations of all formulation species, we tried to keep this effect to a minimum. Based on the design of this study, it remains unclear whether the different buffers are responsible for altering the type and number of formed crosslinks during the crosslinking process (e.g., by influencing the number of formed dityrosine crosslinks or the occurring entanglements) or whether storage medium characteristics cause the differences in the rheological properties by influencing the occurring intra- and intermolecular interactions.

To maintain the hydrophobic ELP constructs in a disaggregated state at room temperature [32] and to allow comparable conditions for all samples, both buffer solutions contain 4 M urea, which causes (partial) protein unfolding for globular proteins and reduces casein micelle formation by disrupting intra- and intermolecular hydrophobic interactions [41,47]. Thus, two effects may occur due to the non-native protein structure, which may be additionally influenced by the different buffer components. First, the altered protein conformation may affect the degree of entanglements, as has been reported for dityrosine-crosslinked ferredoxin-like globular protein [48]. Second, the solvent accessibility of tyrosine residues, and thus the number of dityrosine crosslinks formed, may be altered by the addition of urea [47]. Thus, the addition of urea affects the uncrosslinked proteins either by increasing the solubility of the structurally more disordered casein and the ELP construct used or by inducing protein unfolding of the proteins with an ordered tertiary structure—in this study BSA, which still has a partially stabilized backbone due to the presence of disulfide bridges, and α-amylase. This further influences the hydrogel network formed and the hydrophobic interactions in the resulting hydrogel network. Furthermore, it should be taken into account that high urea concentrations are known to damage cells [49,50], so resulting hydrogels should be washed with physiological buffers to allow applications that require high biocompatibility, such as tissue engineering or drug delivery systems. In conclusion, the formulation buffer has to be chosen carefully depending on the proteins used and the desired mechanical properties. 

### 4.2. Influence of Protein Characteristics

In this study, we used four different proteins that differed in several characteristics, such as their structural arrangement, charge at the pH used, molecular weight, tyrosine content, and more (see Table 1). The successful preparation of hydrogels from all four investigated proteins demonstrates that the applied crosslinking method could be used for a wide variety of proteins to generate protein-based hydrogels and could be applied to more protein constructs. As shown in the literature, the used photocrosslinking approach is known to create covalent dityrosine crosslinks inducing hydrogelation [28,51,52,53]. Since both buffer solutions were prepared with 4 M urea, all proteins were used in a non-native state. Due to the high amount of potential influencing factors and variables which differed for complex protein molecules, no sound statement about the specific influence of the individual properties listed in Table 1 could be made by the presented data. Furthermore, a detailed discussion of the mechanisms responsible for the resulting mechanical properties would require a much deeper understanding of the hydrogel network formed, so this is not addressed in this manuscript. Thus, this manuscript aims to highlight the potential of modulating the mechanical properties of copolymeric hydrogels derived from binary protein mixtures from a more phenomenological point of view.

In the copolymeric hydrogels, the structural strength (G′) was dependent on the interplay of the two proteins used in the binary mixture, without one or the other protein dominating in terms of rheological properties. In this manuscript, we used weight-dependent concentrations of the proteins in mg/mL instead of molar ratios. Thus, the number of protein molecules and their ratio is different for all mixtures. However, this unit was chosen because it allowed the assumption that the total sum of amino acids per ml of precursor solution was comparable. The resulting trend in the rheological properties seems to be related to the type of amino acids, which is further evidenced since the homopolymers derived from different proteins showed different storage moduli.

Considering the elasticity of the hydrogels, the resolution of the analytical method performed does not seem to be sufficient to evaluate the influence of different protein species, as most of the tan δ values obtained were within the standard deviations of another mixture. However, the general trend suggested a correlation and, thus, an interplay between proteins, as seen for structural strength, but this needed to be demonstrated by more sophisticated analytical methods such as nanoindentation or atomic force microscopy. If this correlation was verified in further experiments, this would provide a facile way to tune the rheological properties of protein-based hydrogels based on a low quantity of necessary experimental data. 

While there was no discernible effect on the protein shares of the fracture strain, the compressive strength and hydrogel toughness of binary mixtures were increased compared to the homopolymeric hydrogels for all tested conditions, with the exception of BSA/α-amylase. Furthermore, the structural strength of the homopolymeric hydrogel prepared of casein was more even enhanced by replacing a certain amount of the protein with another protein, which could either be related to inaccurate measurements or a stressed network due to the observed visible cracks in the polymerized hydrogels. Common methods for increasing the toughness of hydrogels include creating more homogeneous hydrogels, introducing an energy dissipation mechanism to limit the propagation of macrocracks (e.g., by interpenetrating polymer networks, fiber-reinforced composite hydrogels, or nanocomposite hydrogels), or combining these two mechanisms [54]. Based on the data presented in this study, it remains unclear which toughening mechanism is responsible for the increased toughness of hydrogel mixtures, as both proteins are crosslinked with the same crosslinking mechanism to copolymers. Thus, to gain an understanding of this effect, it should be analyzed prospectively by advanced characterization techniques such as a profound protein structural analysis before crosslinking depending on the buffer conditions by circular dichroism spectroscopy or Fourier-transform infrared spectroscopy and subsequent hydrogel structure analysis, e.g., by small-angle scattering and dityrosine quantification. For a more detailed overview of different techniques which can be used to understand the nature of crosslinks within hydrogels, please refer to the following review by Fuentes-Lemus et al. [17].

## 5. Conclusions

We have demonstrated the mechanical tunability of protein-based hydrogels by employing binary mixtures of four different protein constructs, either derived from natural sources or recombinantly expressed. The hydrogels were prepared by polymerizing the protein mixtures using the well-developed ruthenium-mediated visible light-induced photocrosslinking method, resulting in a dityrosine formation. Furthermore, we have shown that the rheological properties, such as the structural strength and elasticity, can be modified using binary protein mixtures to prepare copolymeric hydrogels, where the resulting mechanical properties of the hydrogels were found to rely on an interplay between the two proteins used. The copolymeric protein-based hydrogels containing casein exhibited higher toughness upon uniaxial compression than their corresponding homopolymers. The synergistic effects which are responsible for this effect should be further investigated. Overall, the work highlights the feasibility of modulating mechanical properties by simply mixing different proteins with known properties of their formed dityrosine-crosslinked hydrogel network, thus providing a facile way to tailor the properties of protein-based hydrogels to the needs of their specific applications.

## Figures and Tables

**Table 1:** Selected protein characteristics potentially influencing the mechanical properties of the resulting hydrogel network.

Protein	Casein	BSA	α-Amylase	ELP[V2Y-45]
Type	Heteroprotein (consisting of mainly 4 subunits, ≈ 38% α_s1_-, ≈10% α_s2_-, ≈36% β-, ≈13% κ-casein) ^(a)^	Globular protein	Enzyme	Modified protein
Origin	Bovine milk	Bovine blood serum	*Bacillus species*	Recombinant production in *Escherichia coli*
Structural arrangement	No well-defined secondary and tertiary structure of subunits which are forming micelles ^(a)^	Ordered tertiary structure	Ordered tertiary structure	Intrinsically disordered protein
Size/kDa	Subunits: ≈19–25 ^(b)^ Micelles: 250–500 ^(c)^	66.0	52.9 ^(d)^	21.6
Tyrosines/%	≈3.9–4.2 ^(b), (c)^	3.6 ^(e)^	4.2 ^(d)^	6.1
pI (native structure)/-	≈4.6 ^(c)^	≈ 5.0–5.2 ^(f)^	unknown	unknown
Theoretical pI ^(g)^/-	5.0 ^(h)^ 5.3 ^(i)^	6.2 ^(e)^	5.3 ^(d)^	6.8
Theoretical charge at pH 8 ^(g)^/-	−12.6 ^(h)^ −8.4 ^(i)^	−20.9 ^(e)^	−15.1 ^(d)^	−2.0
Adiabatic compressibility (native structure) ^(j)^/Pa^−1^	5.68 × 10^−11 (k)^	10.5 × 10^−11^	5.12 × 10^−11^	unknown
Disulfide bonds/-	Rare, inter-rather than intra-molecular cystine bridges ^(l)^	15 ^(e)^	0 ^(d)^	0

(a) Bhat et al., 2016 [35]; (b) Swaisgood, 2003 [36]; (c) Fox, 2003 [37]; (d) α-Amylase, C8AWK4, UniProtKB/Swiss-Prot; (e) BSA, A0A3Q1LNN7, UniProtKB/Swiss-Prot; (f) Brown et al., 2004 [38] (g) Calculated by Prot pi and ProMoST aspKa database [39]; (h) αs1-Casein, P02662, Uni-ProtKB/Swiss-Prot; (i) β-Casein, P02666, UniProtKB/Swiss-Prot; (j) Gekko and Hasegawa, 1986, [40]; (k) αs-Caseins; (l) De Kruif and Holt, 2003 [41].

## Data Availability

The data presented in this study are available on request from the corresponding author J.H.

## Abbreviations

3D	three-dimensional
APS	ammonium persulfate
BSA	bovine serum albumin
DAC	dual asymmetric centrifuge
ELP	elastin-like protein
ITC	inverse transition cycling
LVR	linear viscoelastic region
MCB	multi-component buffer
pI	isoelectric point
TAPS	N-[Tris(hydroxymethyl)methyl]-3-aminopropanesulfonic acid
MOPSO	3-Morpholino-2-hydroxypropanesulfonic acid
SPB	sodium phosphate buffer
SYMBOLS	
**Greek letters**	
tan δ	loss factor
**ε**_max_	engineered strain at sample fracture in%
**ε_i:280 nm_**	molar extinction coefficient of the protein i at 280 nm
**σ**_max_	engineered stress at sample fracture in Pa
**Latin letters**	
G′	storage modulus in kPa
G″	loss modulus in kPa
n	number of replicates
R^2^	coefficient of determination
